# Relation Medicale de Campagnes et Voyages de 1815 a 1840

**Published:** 1845-01

**Authors:** 


Relation Medicale de Campagnes et Voyages de 1815 a 1840, &c. Par M. le *Baron Larrey*. 8vo. pp. 412. Paris. Bailliere.

No work from the pen of Larrey—the surgeon of a hundred fights—can be devoid of more than usual interest. His vast experience and the practical turn of his ever-active intelligent mind give a force to all his precepts and remarks beyond what we are willing to concede to the majority of writers in these days. Not but that he is too apt to exaggerate the importance of his own views, and depreciate and talk slightingly of whatsoever differs from them; but, despite this very palpable blemish, his writings deserve to be generally known, inculcating, as they do, much that is sound in theory and valuable in practice.

The present volume gives us a rapid review of the closing scenes of his long and memorable career. It has been published upwards of two years; but has hitherto escaped our notice, until very recently. The delay however is not of much consequence; and we therefore gladly take this opportunity of making our readers acquainted with its contents, and of enlivening the *tædium* of purely professional discourse with the amusing anecdotes of an old campaigner's note-book.

In the four volumes of the "Memoires de Chirurgie Militaire et Campagnes," *Larrey* has given a most graphic and instructive description of the leading wants of his chequered life, by flood and field, from the year (1787) when he started as assistant-surgeon in the French navy, down to the first capitulation of Paris in 1814. It is therefore with the view of completing his professional biography that he has brought out the volume which we now propose to consider. The following summary of its contents is given, as nearly as possible, in the author's own words, that the narrative may lose nothing of the amusing gossip of the original.

On my return from the grand army at Fontainebleau, a few days before the departure of Napoleon for Elba, I resumed my very responsible duties as Inspector General of the medical department, and Surgeon of the Imperial Guard. I had proposed to His Majesty to accompany him to the place of his exile; but he graciously refused my offer, saying, "You belong to the army, and you must follow it; it is not without regret that I part from you, M. *Larrey*."

My health at this period had suffered much from the cruel vicissitudes which we had met with in the terrible campaigns of Russia, Saxony and France, as well as from the chagrin I felt at the banishment of the Emperor. For a length of time I was the prey of a gloomy melancholy, which might perhaps have ended fatally, but for the hope I still cherished of seeing once more my illustrious protector. I had indeed formed the project of visiting him in his island, when the unexpected news of his debarkation in France arrived at Paris. He reached Paris on the 20th March; and one of his first acts, on taking possession of the Tuileries, was to summon me to his presence. Alluding to his having left me without fortune, he animated my heart with these words; "Continue your labours, M. *Larrey*, and I yet hope to be able to recompense the sacrifices which you have made and the services which you have rendered to our wounded."

While all was bustle and activity in the metropolis in effecting the re-organization of the army, steps were taken for holding the *fete* of the Champ-de-Mai—a very unnecessary and uncalled-for ceremony under the then existing circumstances. But this, like many other inconsiderate acts of that period, was suggested to the Emperor by the secret triumvirate which had already conspired for his final overthrow. It was about this time that he addressed the Deputies of the department of the Hautes-Pyrenées in the following language, when he presented the new tricolour flags to the different regiments: " Gentlemen, I am pleased to have the opportunity of sending you this flag by your compatriot *Larrey*, who adorns humanity by his bravery and disinterestedness: he has saved a great many of our soldiers in the deserts of Lybia, by sharing with them his little stock of water and spirit, which he needed so much for himself."

As my colleague, Baron *Percy*, had contrived to supersede me at head-quarters as first surgeon of the grand army, I had made up my mind to retire from active duty and devote my time to arranging the materials for the fourth volume of my surgical narrative, when Count *Drouot* waited upon me to invite me, in the name of the Emperor, to accompany him in his campaign, and direct the *ambulances* of the guard. I could not resist the appeal, and at once prepared to depart. We left Paris on the 9th of June; but—at least in my own case—not with those feelings of confidence that used to animate me in all our former expeditions. My mind was haunted with the presentiment that this campaign would prove disastrous to France. The sight of one of our grenadiers, on the evening of our first day's march, stretched out in a corn-field by the wayside with his head shattered to pieces, added to the gloom of my ideas, and it was not without the worst forebodings that I continued my route towards the frontier.

On the 16th we fought the battle of Fleurus (Ligny)—the scene of Marshal *Jourdan's* famous victory in the days of the Republic. The result was not decisive; either from our not profiting, to the full extent, from the advantage gained, or from the Emperor being diverted from following up his success with his usual promptitude and decision by persons around him who were interested in his overthrow. While our army rested for twenty-four hours after this conflict, the enemy was enabled to rally their forces and resume an advantageous position. On the morning of the 18th, they were discovered along the line of Waterloo. The battle on this field had not long been joined, before the Prussian and English columns gave signs of confusion and retreat; but, at this time, torrents of rain came down and materially impeded the operations of our artillery, the ground becoming so soft and miry that our gun-carriages could not be moved. The agents of the traitors that existed in our own army, as well as of the enemy's officers, taking advantage of this *contre-temps*, spread themselves among the ranks, calling out to the artillerymen, " Save yourselves who can; cut the traces of the horses and be off, for you are surrounded on every side by the columns of the enemy." An eye-witness of these treacheries, I longed to acquaint the Emperor of them; but the wounded on every hand were calling out for my assistance, and, in addition to this imperative duty, I had to look after my *ambulances*. The enemy's cavalry now made repeated and most destructive charges upon our lines, and their daring was not a little increased by their knowing full well that ours were at a distance from the scene of action. Marshal *Grouchy*, with his 30,000 troops, had remained at a distance from the main-body of our army, which had consequently to resist the entire combined forces of the coalition. The disorder therefore increased more and more; and the wounded came in so fast to us on all hands, that, in spite of our best zeal and courage, our efforts were paralysed, either from the charges of the English cavalry reaching even to our *ambulances*, or because we were deprived of the light of day. We were therefore forced to join in the retreat which our army had begun to execute, according to the instructions which I had received from the Emperor by an aide-de-camp, to gain the frontier by a cross-road that was pointed out to us. We had not proceeded above two or three leagues when, in the dark, we were overtaken by a troop of the Prussian lancers. I was marching, at the time, at the head of my small company, and, feeling convinced that our assailants were not numerous, I did not hesitate, sword in hand, to force my way through them. Discharging my two pistols, I made an opening, through which my companions and servant passed, and galloping off at full speed, contrived to gain the open plain. My horse being wounded, fell; and at the same moment I received a sabre blow upon my head and left shoulder, which brought me senseless to the ground. The troopers rode after my companions, and overtook them. Meanwhile, having gradually recovered my consciousness, I got up, mounted my horse, which had by this time revived considerably, and made my escape through the corn-fields, directing my course towards the frontier. Next morning, by the break of day, I reached the banks of the Sambre, and was immediately made prisoner by the enemy's cavalry. The soldiers treated me very badly; for they stripped me not only of my arms, watch, and all my cash, but also of my very clothes. My height, and the grey great-coat which I wore, made them mistake me for the Emperor; and, under this idea, I was conveyed without delay, handbound, to the Prussian general in command of the advance guard. He soon discovered the error; and, in a fit of fury, determined to have me shot. But at the moment when the soldiers were ready to shoot me in the head (faire feu sur ma tete), the surgeon of the regiment recognised me, and prevented the execution of this barbarous order. I was forthwith taken before General *Bulow*, who knew me, and had compassion on my miserable condition; for at this time, besides being barefoot and having scarcely withal to cover my nakedness, my hands were tied behind my back, and my head was covered with bloody rags. When Marshal *Blucher*—to whom my name was well known, as I had saved the life of his son who had been wounded and taken prisoner in one of the Austrian campaigns—heard of my state, he treated me with great generosity, and sent me on with one of his aides-de-camp, to Louvain, where I was lodged in the house of a poor widow. A young *officier de santé* was summoned to dress my wounds. He at once recognised who I was, exclaiming, "You are Baron Larrey;" and forthwith, before I had time to answer him, he hurried down the stairs and disappeared. He had gone to announce my name to the municipality of the town, and to obtain, if possible, better accommodation for me. In a very short time my young medical friend, accompanied by one of the civic officers, came back; and I was conveyed in a carriage to the house of

M. *Yonk*, a distinguished advocate, from whom I received all manner of kindness. Every where the Belgians appeared to sympathise in heart and feelings with the French, and, I doubt not, they very sincerely regret that they have not been rejoined to this great family.

After a few day's care and quietude, I was able to visit some of the hospitals into which many of our wounded soldiers had been admitted. At this time, I was much too weak to operate myself; but I assisted with my advice the Belgian surgeons in the performance of their arduous and responsible duties: their zeal and intelligence merit my warmest praise.

From Louvain I passed on to Brussels, where the English had established their hospitals. During the first few days after the terrible battle that had been fought on the adjacent field, it had not been possible to class or arrange the wounded of the different nations; and hence they were all confusedly mingled together. Most of the French however were in the military hospital of the town, under the immediate care of my old fellow-labourer, Dr. *Seutin*. We performed together a number of operations, most of which terminated very favourably. Occasionally I visited the English hospitals, and consulted with the surgeons on the more difficult cases. There was one case that I took especial interest in, and which merits a short notice here. A French soldier had his right thigh so terribly disorganised by a cannon-ball, that the only chance of saving the poor fellow's life was by amputating the limb at the hip-joint. This formidable operation was successfully performed by Mr. *Guthrie*, and the man is now an inmate of the Hôtel des Invalides.

I obtained permission from the allied officers to return to my family; and straightway I set out for Paris, where I arrived on the 15th of August. All the barriers and military posts of this city I found occupied with foreign troops—a woful sight to one whose soul was already so deeply wounded by personal misfortunes. New troubles awaited me! Regarded as one of the most zealous partizans of *Napoleon*, I had been deprived of my office as inspector-general of the army, and of all its emoluments. Besides this loss, I ceased to receive any pay as member of the Legion of Honour. My family too was *oberée* by having foreign soldiers billeted upon them, so that altogether my ruin appeared to be complete.

But even then I was not without some prospects of advantage. Not only was I solicited to pass over to the United States, but an offer of rank and high responsibility in his army was made to me by the *Emperor Alexander*. *Don Pedro* of Brazil also wished to engage my services for his army and as professor of surgery in the University of Rio-Janeiro. But I was resolved not to expatriate myself, although exposed both to distress and even danger during the counter-revolution that was secretly at work during the years of 1816 and 17. On the following year, the pension of 3000 francs—which *Napoleon* had bestowed on me for my services after the battles of Wagram, Lutzen, Bautzen, &c.—was restored to me by a decree of the Chambers. By this act of grace, my difficulties were considerably relieved, and I was enabled to carry on the publication of the fourth volume of my *Relation*, which was received with much favour by the public.

In 1822 the news of the death of the Emperor overwhelmed me with affliction, and, partly with the view of drawing my mind away from melancholy recollections, I commenced a work on Clinical Surgery, which I had been contemplating for some time. Before, however, completing it, I resolved to pay a visit to the hospitals in England—one of the most civilized nations of Europe and where Surgery had made, of late years, such brilliant progress—to examine for myself the operative practice of the surgeons there, and compare it with our own. Another motive with me was to take my son Hippolyte, then 18 years of age, with me, in order that he might complete his knowledge of the English language; for I had been long convinced that nothing contributes so much to enlighten and expand the mind and stimulate it to exertion, as travelling.

Having obtained from the Minister of War official permission to go abroad, and retain, during my absence, *ma solde de traitement*, we started from Paris on the 26th of August, 1826. Passing through Rouen we proceeded to Havre, where, among other sights, we visited a French frigate, which had just returned from the West Indies. I was much struck, and not a little pleased, with the great improvements that had been effected in the general economy of our ships of war since the year 1787 and 1788, when I served in the Royal Marines. The substitution of dry-rubbing of the timbers between decks for the use of the wet mop is of essential advantage to the health of the crew; and the preservation of the water in iron tanks in place of wooden casks has likewise been most beneficial. The water might be kept still sweeter than it is, if the tanks were lined with tin, and their walls were rubbed over with peroxide of manganese.

The clothing of the sailors, too, in the present day, has been much improved: the woollen shirt, that they wear next the skin, contributes much to the preservation of their health, alike in hot and in cold climates. The hammocks, also, are made much more comfortable than they used to be at the close of the last century.

Arriving at Portsmouth, the Baron went on board the Victory—*Nelson's* ship—with which he was quite delighted. His notice was especially drawn to the famous words—engraven in letters of gold on the quarter-deck—of the last signal made to the fleet on entering into action at Trafalgar; "*L'Angleterre devait s'attendre qu'aujourdhui, ici, chacun ferait son devoir.*" (How feeble, when compared with the original!)

He visited the Naval Hospital, and expresses his admiration of the arrangements, both medical and administrative, of its entire economy.

Journeying through the West of England, he first passed over to Dublin, the various medical institutions of which he examined with great care.

The following remarks, on the treatment of maniacal Insanity, seem to have been suggested by what he saw in the great Lunatic Asylum of that City. "In place of using cold *douche* baths, in which the water is made to fall from a considerable height on the shaved head of the patient—a method that is both painful and apt to induce hypertrophy of the cranium and engorgement of the cerebral membranes, a common cause of the incurability of insanity—I should advise the application of ice to the vertex. Bleeding, either general or local, should generally precede the adoption of this remedy. The subsequent use of revulsives and antispasmodics is often of much service." The Baron tells us, that his estimable Irish *confreres* received these, and all his other suggestions, with every mark of grateful respect, and promised to adopt them in their practice.

He comments, apparently in a spirit of disapproval, upon the custom, in this country, of applying the ligature to the femoral artery, in cases of popliteal aneurism, so high up in the thigh as two or three inches below Poupart's ligament; and, also, on the great unwillingness of British surgeons generally to have recourse to the use of the trephine in injuries of the skull.

The Foundling Hospital of Dublin is regarded by our traveller as altogether more beautiful and more admirably conducted than any similar establishment in Europe: *il fait honneur à la generosité et à la philantropie des administrateurs qui le gouvernent.* The Maternity Hospital also, of this metropolis, deserves the very highest praise.

*Larrey,* like every other visitor of Ireland, could not fail to remark the miserable condition of a vast proportion of the lower classes of the population. Their squalid unclothed condition is truly lamentable. Little is the wonder that Typhus fever is so prevalent and destructive in places that are unfortunately so well prepared for the development and spreading of this pestilence. The fever here is often accompanied with an erythematous exanthem, and sometimes even with carbuncular pustules, not unlike to what I had seen in plague-patients in Egypt. The treatment, continues our author, that was followed in this disease, appeared to me to be most rational, and on the whole very successful. Revulsive bleedings, from the nape of the neck and from the back, with the cupping instruments, and the internal use of etherial theriacal wine, of bark or quinine, and of lemonade, constituted the basis of the medication. I suggested, as a very efficacious remedy in all such cases, the application of the moxa near the basis of the cranium and on the epigastric region.

For opening the sympathetic or critical abscesses, that not unfrequently supervene in Typhoid patients, I advised the application of the actual cautery—the point of a knife heated to whiteness—or of the caustic potash, in preference to the use of the bistoury, that appeared to be used in this country on all occasions. I also proposed to my medical friends to extirpate the malignant pustules or carbuncles, and then to cauterise the wounds with the heated iron! This, like every other observation, was received with 'une grande modestie et d'un veritable interêt.'

The illustrious stranger met with similar attention from his professional brethren in Liverpool and Glasgow, to whom he pointed out the proper method of applying his unremovable apparatus for the cure of fractures—the treatment of which, among us, he considered to be decidedly faulty—and also the moxa, as improved by him.

*Larrey* arrived in London from the North on the 14th September. "We were," says he, "singularly astonished and almost awe-struck (effrayés) at the magnitude of this city." One of the first visits he paid was to Sir *Astley Cooper,* who, as we may well suppose, received his visitor with distinction and all the marks of a sincere fraternity. They drove together to Guy's, which, like all the other hospitals in England, is admirable for its cleanliness and excellent arrangement.

He then went to Chelsea Hospital, in order that he might compare the condition of its inmates with those of the Hôtel des Invalides: the latter he considered to be, on the whole, both better fed and better clothed than the former. The pensioners of the English army are lodged in small apartments, separated from each other, and arranged like the cells of monks. We were agreeably surprised to see a plaster statue of Napoleon over the door of more than one of these. Sir *Everard Home* and Dr. *Somerville* paid all attention to their illustrious guest; the former presented him with a copy of his magnificent work on Comparative Anatomy.

In reference to what he saw at Chelsea and Greenwich Hospitals, the Baron makes the following remarks, inculpatory of English military surgery in reference to amputation. "We found in these establishments only a very few patients who had lost their limbs. One of the principal causes of this circumstance, as I had already observed during the course of the war, is the unsuccessful result of the amputations in the practice of the English surgeons—a result which appears to me to be attributable to the mode of operating and the plan of dressing the wound that are almost invariably followed. These processes consist in making the amputation, or rather the section of the muscles, *en un seul temps*, and in uniting the flaps of the wound too firmly together with the view of obtaining a cure by the first intention. A long experience has quite satisfied us of the disadvantages of such practice."

After inspecting the various medical institutions of the Metropolis, *Larrey* visited the British Museum, where "he saw with genuine pain and regret the precious and rare antique monuments which we (the French) had collected in Egypt, and which are figured and described in the great work commemorative of our brilliant expedition. The most remarkable of these monuments are the famous stone of Rosetta, on which is graven an inscription in four languages—the Hieroglyphic, the Coptic, the Arabic and the Greek—and the closed hand, in red granite, of the Memphian Colossus, which we found buried at a considerable depth in the soil of the ancient capital of Egypt. The first phalanx of the middle finger of this hand I measured at the time of our discovering it, and found it to be three feet long. Judge then the enormous state of the entire figure! Besides these, I saw many other curiosities which the English had taken from us at Alexandria."

The Baron expresses his regrets that the urgency of his duties prevented him from a longer sojourn in London. He had seen much to interest him, and he left with every feeling of gratitude and respect.

"I remarked with satisfaction," says he, "that the English surgeons perform their operations with perfect calmness of manner, and with great dexterity: they do not hurry or make a bustle with their instruments, as I have seen a good number of our distinguished surgeons do.

"I cannot give the same approbation to their plan of dressing the wounds after their operations. Their great aim and object seem to be to cause the solution of contiguity to disappear. The result of this over-anxiety is that not only they bring together and place in exact apposition the edges of recent wounds, but they even treat old wounds and ulcers in the same manner.

"In general, it is a physiological error to believe that we abridge the labour of nature in the cicatrisation of wounds, by bringing and retaining their surfaces into immediate contact with each other, when the wound is the result of amputation of a limb in its continuity. In this case it should be remembered that all the tissues or organs are divided perpendicularly to the axis of the bone. However exact may be the union or apposition effected at the time of the first dressing, there must always remain at the bottom of the wound a cavity in which will accumulate the fluids that continue for some time time to ooze out from the open small vessels. In this maner, therefore, there can scarcely fail to be a more or less considerable effusion, the presence of which must necessarily prove injurious, on the one hand by the distention and tumefaction of the two flaps thereby occasioned, and on the other, by the absorption of a perhaps putrid matter into the circulation, and a consequent infection of the whole system. The presence of pus in the veins of an amputated limb is no proof of the vessels being inflamed; they are only the conductors of the morbid matter generated in the wound. Even the divided nerves become penetrated by the miasmatic principle, or by the carbonic acid developed in the purulent *foyers* that are formed in consequence of a sort of fermentation.

"When the tissues of an injured part are in a state of inflammation, they are not at all in an apt condition to unite; and the only way, in which they can become *degorged,* is by the process of suppuration being established."

Our author discourses for several pages on this subject of primary and secondary union of wounds after amputation.

"When the operation is performed for any chronic disease of the extremity, as for caries of the bones, white-swelling of the joints, or cancerous or other malignant degeneration of the soft parts, I consider it to be quite indispensable, if we hope to prevent local engorgement and purulent infection, that the divided surfaces be not immediately united and kept together; so that the effused fluids may not be retained or forced back upon the system, but may ooze out from the wound as they are formed. Nearly the same remarks hold good in cases where a limb has been amputated for a recent injury. At least, such we have found to be the case in our own experience; and the surgeons of our army at the recent capture of the Citadel of Antwerp fully confirmed the truth of my remarks. In almost all the cases, the immediate union had been adopted at the first dressing of the stump; but, within 12 or 24 hours, the re-action which ensued was found to be so violent as to compel the surgeon to remove the bandages and plasters. On this being done, the unpleasant symptoms subsided; but in not a single instance was the healing complete before the 50th or 55th day after the operation. In the cases, on the other hand, that were treated on my principles, the wound had generally healed full three weeks sooner. In a very few instances, the exact and primary union took place, and the healing of the stump was very rapidly *(prematurement)* effected: but what usually happens then? The internal adhesion does not and cannot take place except by a sort of mere *recollement* or agglutination of the divided tissues, so that the vessels cannot be retained in such a mutual apposition as to anastomose with each other, and the divided extremities of the nerves cannot unite together, end to end, as constantly happens when the treatment of the wound has been conducted on my principles, and according to the obvious intention of nature, which will never be violently thwarted by the intelligent surgeon. Moreover, the stumps, that are prematurely cicatrised, generally remain more or less painful; moreover, they are apt to ulcerate on the slightest causes, and even sometimes to mortify. * * * * In fine, Nature must work slowly and without disturbance to effect the process of healing the wounds after surgical operations, whether these have been performed for chronic diseases or for recent injuries. This department of surgical practice in England has always appeared to me to be far from the perfection which might be expected from such able men as are the surgeons of that country."

So much for the alleged superior advantages of secondary over primary union after amputations. *Mais il faut nous mettre en route.*

M. *Larrey* left London at the end of September, and proceeded to Chatham, where Sir James Macgregor received him with great kindness, and shewed him every mark of courtesy. He was much pleased with the beautiful order and cleanliness of the hospital at Fort Pitt. He approved of the practice of having the bedding in all the beds folded back during the day, so that the soldiers could not be lying down upon them. The military discipline appeared to him to be very rigid and even severe; but the men, he allows, were well fed and clothed; and each one had a bed to himself. (We infer that such is not the case in the French hospitals.) *Larrey* then accompanied his host to the mess-room, where a handsome collation awaited him, and the health of "the surgeon of Napoleon," was drunk with great enthusiasm. "On leaving the garrison, military honors were paid to me, as to a general officer—a mark of distinction which alike surprised and delighted me. Altogether, the visit to this place was so gratifying to my feelings, that it can never be effaced from my memory."

When he arrived in Calais, he found awaiting him a summons to Picardy to visit General *Caulincourt*, Duke of Vicenza, who was lying dangerously ill. Thence he went on to Paris, to resume his official duties at the military hospital of the Royal Guard, and to complete several projects which he had in hand. Among other things, he submitted a report on the state of the barracks and hospitals in England to the Minister of War, General *De Caux*. He engaged also in the publication of his *Clinique Chirurgicale exercée particulierement dans les Camps et les Hôpitaux Militaires, depuis* 1792 *jusqu'en* 1829—in which year he was elected a member of the Institute, on the death of his preceptor, Professor *Pelletan*. He had long been ambitious, he tells us, of this "glorieuse admission;" and at length his desire was attained.

In the course of the following year, 1830, so memorable for important events, happening to be upon a jury, when a handsome and well-educated young man was tried and convicted of forgery, and thereupon condemned to be *branded* and sent to the hulks for ten years, the Baron was so afflicted at this barbarous sentence—of the shoulder being branded with a hot iron by the common executioner,—that he made an appeal to the royal clemency to have it remitted in the present instance. The favour was granted; and before many months were over, I had the satisfaction, adds he, of finding that this article of our criminal code was expunged on the establishment of the present dynasty in France. During the Revolution of this year, I had a serious, and sometimes even a dangerous, part to perform in the execution of my official duties. The Royal Guard, as the main prop of the Royal cause, were especially obnoxious to the people; and, on the third day of the fighting, a vast mob had collected and determined to break into my hospital and wreak their vengeance against the wounded inmates. I threw myself amongst the assailants, and addressed to them a few forcible words, appealing to their honour and sense of pity in behalf of my poor patients. The result was that they dispersed, having first made themselves masters of all the soldiers' fire-arms. Having been subsequently appointed a member of the medical commission to report upon the wounded of the three days, I received, as a reward of my services, the Decoration of July from the hands of his Majesty King *Louis Philippe*.

In the latter part of this year I was invited by King *Leopold* to Belgium, to organise the *ambulances* of his army, and to inspect the hospitals in different parts of his new country. My attention was also very particularly directed to the study of the Epidemic Ophthalmia, which had already struck with blindness a great number of the Belgian troops.

The hospital at Antwerp I found to be very faulty in its construction and general management. The ophthalmic cases, amounting to fifty at least, were all assembled together in an ill-ventilated dark ward on the ground floor, the unwholesomeness of the place being not a little increased by there being close-stools for the use of the patients! Upwards of thirty of these poor fellows were entirely blind.

The worthy Baron here enters upon a brief digression to shew that the French soldiers are on the whole infinitely superior, not only in physical force but also in intelligence and moral energy, to the Dutch, Belgian and German troops. We did not for some time perceive the object of introducing this patriotic comparison here, till we came to a passage where he alludes to the stolid indifference of the Belgian blind soldiers under their bereavement, and contrasts it with the fierce despair of the French, many of whom in Egypt, he tells us, threw themselves into the Nile, or put an end to their lives with their fire-arms!

He strongly condemns the practice that was pursued by the Belgian surgeons in the treatment of this Ophthalmia. After the application of a few leeches to the temples, a collyrium, containing some corrosive sublimate and extract of belladonna, was generally used. "The effect of this application was to blunt the nervous sensibility of the eye, to paralyse the retractile or elastic action of its vessels and membranes, and consequently to keep up the *stasis* of the fluids in the deep-seated tissues. Besides these injurious results, the tissue of the cornea often became thereby shrivelled and crisped, so that ere long it was rendered more or less completely opaque, and insensible." He advised that all the patients should be immediately sent to more healthy quarters, and that the wards should be well lime-washed: he also pointed out the mode of treatment (it is not specified) which he had found most useful in his own experience in Egypt and elsewhere. The condition of the military hospitals at Louvain and Liege was no better than he had found at Antwerp: the wards were unwholesome, and the soldiers, besides everlastingly smoking, were often in a state of "malpropreté repoussante." The medical treatment of the ophthalmic cases was the same here as what we have already alluded to. In the report, which subsequently he sent in to King Leopold, we find the following passage, that deserves especial notice.

"My enquiries respecting the health of the troops of your army have convinced me that the ophthalmia, which is so wide spread and so destructive, is by no means contagious in its nature, as many of the medical men suppose; and that it has been produced exclusively by certain causes of insalubrity to which the soldiers are exposed; as, for example, by their not guarding themselves against the humidity of the nights, by their excessive smoking even within the barracks and hospitals, by the immoderate use of spirituous drinks, and by the neglect of cleanliness."

On his return to Paris, *Larrey* was appointed head surgeon of the Hôtel de Invalides, of which Marshal *Jourdan* was then the governor. France was at that time threatened with an invasion of the Oriental Cholera; the disease having already made its appearance in Germany and Britain. The pestilence was not long in reaching Paris, and the Hôtel was one of the places that was first attacked. A good many deaths took place within a short time among the inmates; but the Baron was satisfied, after a minute examination of the subject, that the malady did not propagate itself by contagion.

He seems to have been very anxious at this time to have introduced a good many changes into the general economy, as well as the medical management, of the old soldiers; such as establishing a set of lectures, musical concerts and other modes of recreation among them; but his zeal appears to have somewhat outrun his discretion, as none of these changes were allowed by the administrative council. We should think that the worthy Baron must on a good many occasions have given offence to his medical brethren also, by his almost invariable censure of their practice, and his frequent and excessive laudation of his own. We must however make some allowance for the garrulous egotism of old age and long service.

In September 1834, he started with his son on a tour to Italy, inspecting on his way the military establishments in the South of France. At Genoa he first visited any of the foreign hospitals. The surgical practice pursued there did not meet with his approbation: "elle est eloignée des bons principes et depourvue de toute methode."

A visit to the beautiful cemetery of Leghorn the *Campo-Santo*, where so many strangers from the North—the victims of phthisical disease—are interred, suggests the following very sensible remarks:

"It is a very fatal error, or perhaps rather a very pernicious prejudice, for medical men to imagine that mild and warm climates can effect a cure of thoracic diseases. The cold and humidity, which generally are the determining causes of these affections, doubtless aggravate their symptoms; but, when the mischief has reached the second stage of its career, atmospheric heat almost always does harm instead of good, and very generally accelerates the fatal issue. It is the same with phthisis as with all chronic maladies that are attended with hypertrophe or engorgement of the parenchymatous or membranous viscera."

Our author goes on to point out his method of treatment in these and other organic diseases—induced, according to him, by the presence in the system of a peculiar virus or morbific principle. (!) His chief remedies seem to be mercury and the use of the *moxa*—by means of which he has effected, he assures us, many most marvellous cures: *experientia probat*.

The number of monuments in the cemetery of Pisa over the foreign victims of chest-diseases is even greater than at Leghorn; and yet a residence in the former city is especially recommended by many physicians in such cases!

At Rome he visited Cardinal *Fesch*, who introduced our traveller to his sister, Madame *Letitia*, the mother of *Napoleon*. She was at that time eighty-eight years of age and entirely blind. Her reception of the faithful companion of her illustrious son was most affectionate. His report on the state of surgical and medical practice in the hospitals of the Eternal City is far from being favourable. According to him, the use of cupping is scarcely known; at least, this most valuable remedy seems to be never employed, in Italy. "I had," says the Baron, "great pleasure in shewing some of my professional brethren in Rome the manner of practising this method of revulsive bleeding, and also the application of the moxa, according to my plan."

Syphilis is exceedingly common in Rome; but the disease is usually not very severe. It is almost always treated with mercury, administered internally, and rubbed on the soles of the feet. It is truly a melancholy sight to find in the wards of the hospitals girls of ten, and even of eight, years of age, labouring under this disgusting malady.

The Baron was much struck with the prodigious number of ecclesiastics in modern Rome. Their number is said to amount to not fewer than 25,000—more than a sixth part of the whole population. They are usually of a plump, and healthy-looking exterior. In all the convents, there is a *pepiniere* of children; and these are usually attired in the dress of monks. The vast number of beggars too excited feelings of disgust and regret, wherever he turned his eyes. The lower classes of the Roman population reminded him a good deal of those whom he had seen in Dublin; the same contrast between the opulence of the few, and the miserable destitution of the many, is too often painfully striking in both cities. "Every thing languishes in Rome. The Papal government does nothing to rectify abuses, to correct prejudices, to arrest the destruction of the very monuments which form the country's glory, or to render the place more wholesome. Industry is utterly unknown; cities decay; and the people are becoming more and more degraded. Had *Napoleon* lived, this, the second city of the French empire, would have been alike reformed and embellished."

Our traveller returned from Rome to France through Florence, where he was hospitably received by Prince *Louis Buonaparte*, the ex-king of Holland, his sister *Caroline*, the widow of *Murat* and ex-queen of Naples, and his sister-in-law, the ex-queen of Spain.

He inspected with great care the hospital establishments at Toulon, where the chief surgeon, M. *Reynaud*, shewed him every mark of attention. He saw, in one of the wards of the marine hospital there, a fractured leg placed in *Mayor's* suspended board: he strongly disapproved of this apparatus, and recommended his own "appareil inamovible." At Aix, the hospital, he tells us, is large and beautiful; but the surgery, that is followed, is that of the 17th century. Among other cases, he severely criticises one of amputation of the right leg *immediately above the ankle-joint*. The stump was left pointed and conical, and the limb was always in a half-bent position, so that the patient could scarcely make use of any sort of wooden leg. This operation should never be performed: the preponderance of power in the flexor muscles over that of the extensors must always prove an impediment to a patient being able to walk with the stump introduced into the case or *etui* of any mechanical leg.

At the Hôtel des Invalides at Avignon,—of which General *Lenoir*, who lost a leg in Russia, is the governor—the Baron saw a number of his old comrades of the Imperial Guard, who, he was delighted to find, were well cared-for in every respect.

He had not been long in Paris, when he was called upon by Marshal *Maison*, the then minister of war, to repair to the South of France, where the cholera had made its appearance, and was occasioning the greatest panic. He accordingly started on the 21st July, 1835, for Marseilles, and arrived there in three days afterwards. The inhabitants, in this and the other towns in which the epidemic had broken out, were in a state of the utmost alarm. Crowds of people had left their houses and were taking refuge in the fields. Even the medical men had partaken of the general trepidation; for no one, we are told, had been bold enough to examine the body of a patient who had died of the disease, till the Baron set them the example. The priests too had, in his opinion, contributed not a little to increase the general alarm, by getting up religious processions, in which the holy relics of the different churches in the place were paraded about the streets, with the view of deprecating the Almighty's anger. *Larrey* takes this opportunity of expressing his regret, that *Napoleon's* intention to confine all religious ceremonies to within the walls of churches, was not in force at the present day, throughout the country. In the fear and confusion that everywhere prevailed, the bodies of the deceased were often buried within three or four hours after death. On one occasion it was alleged that a woman had been interred before she was quite dead. This circumstance produced the greatest excitement; and had it not been for the Baron's suggestion, that every dead body should be covered over with a sheet that had been immersed in a solution of chloride of lime, and that no funeral need take place within twenty-four hours after death, there would, he says, have been a popular tumult. By his promptitude and zeal, he succeeded in effectually quieting the apprehensions of all classes.

*Larrey* seems to have regarded the morbific principle of Cholera as of an animalcular nature; and he talks of the disease as a "neurose ataxique," evidently regarding it as allied to certain malignant fevers. He peremptorily denies its contagious character; but he admits that its diffusion may be much promoted by want of cleanliness, tainted air, &c. His treatment consisted in the use of ipecacuan emetics in the first instance; in frictions of the surface with snow, or, in defect of this, with camphorated oleaginous or spirituous embrocations; in the administration of ice, and of light aromatic infusions; and subsequently in the application of the cupping-glasses—with or without the scarificators—to the hypochondria, epigastrium, abdomen and back, and afterwards of sinapisms and moxas to different parts of the body. He disapproves of almost all the internal remedies that have been recommended, and reprobates with especial severity the practice of injecting fluids into the veins. But it is unnecessary to pursue this subject. On his return to Paris, he made an elaborate report upon the epidemic, and received the thanks and approbation of the Commander in Chief. In 1841, *Larrey* went over to Algeria to visit the military hospitals in that new colony. Immediately upon his return from this expedition, he was taken ill and died at Lyons. In a former Number (for January, 1843) of this Journal, we have given an account of the funeral orations that were pronounced over the illustrious deceased: to that article we may refer for some further particulars of his life and services.

In an appendix to the present volume, we are presented with a "Statistique Chirurgicale," or series of short notices of the most distinguished of our author's patients, prefaced by these words:—"The history of their wounds may, I think, occupy a few pages that will prove not without interest or value for the study of those memorable wars which

France had to sustain against all the powers of Europe, from 1792 to 1815." We shall select the most interesting of these notices.

General *Almeras* was severely wounded at the battle of the Pyramids. A musket ball traversed the pelvis from before backwards, passing between the testicles, perforating the neck of the urinary bladder and also the rectum, and escaping from the inner part of the right hip. In spite of the dangerous nature of the wound, this brave officer recovered, and eventually served in the campaigns of Germany, Russia and France. This case is certainly one of the most remarkable that I ever met with in practice.

General *Andreossy* suffered severely from the endemic ophthalmia in Egypt; but recovered perfectly. He died in his native town of Castelnaudari.

General *Arrighi*, Duke of Padua, was wounded in the neck by a musket-ball at St. Jean d'Acre. The right carotid artery was wounded; and he must inevitably have died of hæmorrhage if a soldier had not had the presence of mind to introduce his two fore-fingers into the wounds, until I arrived and tied the vessel. During the operation, a shell exploded over our heads; but, by a sort of miracle, none of the fragments struck us. The recovery was complete and the General is still alive.

Prince *Eugene Beauharnais* was wounded by a ball in the right temple, at the seventh assault of the same fortress, St. Jean d'Acre. General *Bertrand* too was similarly wounded at the first battle of Aboukir. It was on this occasion that *Napoleon* first became acquainted with this most faithful and devoted of his servants.

Marshal *Bessieres*, Duke of Istria, received a severe contusion of the thigh in the celebrated battle of Wagram: his horse was shot under him at the time. This great warrior was subsequently killed by a cannon-ball, while reconnoitring the enemy's lines on the evening before the battle of Lutzen.

General *Blaniac* received, at Aboukir, a gun-shot wound in the right side of the chest. The ball had passed from before backwards, following the direction of the third sternal rib to the seventh, which was fractured in its posterior third: the middle lobe of the lung was injured, and the intercostal artery lacerated. There was a good deal of hæmorrhage from the anterior wound. Symptoms of sanguineous effusion into the chest subsequently occurred, and the operation of *paracentesis thoracis* was performed by enlarging the wound behind. Upwards of a *litre* of dark-coloured fluid was discharged. By the most assiduous care, continued for three or four months, this meritorious officer quite recovered his health.

The only real wound that General *Buonaparte* (so he is styled here by our author) ever received in his long career of danger, proceeded from a kick of the first Arab horse which he mounted, on leaving the Desert of Lybia. The contusion was followed by an extravasation of blood, to which I gave vent by a small incision. The wound quickly healed. (We have surely read somewhere that *Napoleon* received, in one of his Austrian campaigns, a contused wound of the leg, that proved, however, to be of no great severity, although great alarm was at first occasioned in the army by the rumour of it.)

General *Caffarelli* had his right elbow shattered at the siege of St. Jean d'Acre; he was overturned by the blow and fell heavily on his right side. The arm was immediately amputated, and everything went on favourably till the 21st day, when symptoms of hepatitis made their appearance. A few days afterwards he died. On dissection, several large abscesses were found in the liver; one of these had burst into the abdominal cavity. The suppurative inflammation had doubtless been induced by the fall.

General *Champeau* was severely wounded by a cannon-ball at Waterloo. Before I could reach him, a sudden charge of the English cavalry obliged me to withdraw to a considerable distance; and the poor wounded man, like many others, died on that disastrous field without relief.

General *Cheminau* had his right leg disorganised by a cannon-shot, at the battle of Lutzen. Although the injury of the soft parts and bones extended close up to the knee, yet, as the joint itself appeared to be intact, I determined to amputate the limb immediately below it. Having made a flap, I disarticulated the head of the fibula, and sawed the tibia across, directly below the attachment of the capsular ligament. I was then surprised to find that the two condyles of the bone were separated by a vertical fracture: the ligament, however, remained uninjured, and there was no symptom of extravasation within the joint. A uniform and circular compression was maintained around the condyles of the tibia, and the dressing of the stump was finished by the application of my unremoveable bandaging. This was not removed for nine days, and then immediately re-applied. The success was complete.

General *Coutel*, commanding the company of the aeronauts attached to the army of Egypt, and who, by means of ascending in a balloon, had ascertained the position of the enemy's army before the celebrated battle of Fleurus, received a musket-ball in his right arm at St. Jean d'Acre. Although the humerus was broken, and the injury of the soft parts severe, I determined to try to save the limb. The ends of the bone did not, however, unite, and a false articulation was formed.

General Count *Daboville*, now a Peer of France, was wounded most severely in the right shoulder at the terrible battle of Wagram. This was one of the fourteen amputations at the shoulder which I performed on that day! Of these cases, twelve recovered perfectly; one patient was killed accidentally by being thrown out of the *ambulance*; and another died of hæmorrhage during the subsequent evacuation of Vienna.

General *Damas* was killed by a cannon-shot which struck his chest, at the battle of Moskowa. He was one of the 40 general officers who either fell or were most severely wounded on that dreadful day.

General Count *Danthourd* was wounded at the battle of Pesth (1805) by a musket-ball, which, after breaking his spur, doubtless penetrated into his right tarsus, and there lodged deeply. Imagining that this wound was only a flesh one, he applied a piece of wetted rag to his foot, and straightway continued his march to the field of Austerlitz, at the glorious battle of which he was present. His wound healed; and he experienced no inconvenience in the foot until 15 years afterwards, when he began to feel sharp pains in a tumor, as big as an olive, which was situated on the dorsal region of the tarsus. Two of the leading surgeons in Paris considered it to be an exostosis; and the General himself was not aware that any ball had ever lodged in the part. On his shewing it to me I soon recognised what it was, and told him how he might get rid of it. The ball on extraction proved to be angular, and was lodged in a fibrous cyst.

General *Desaix* was wounded, in one of the combats before the capture of the lines at Weissembourg, by a ball which perforated his left cheek. He was killed at the battle of Marengo, " dont il fixa la victoire."

The Duke *Deselignac* received a gun-shot wound in the left foot and ankle-joint in the last combat of July 1830. The limb should unquestionably have been amputated at the time; but this was not done, and, on the seventeenth day after the accident, symptoms of tetanus supervened. On being called into consultation, I recommended that the limb should be amputated: but this proposal was not acceded to by the other surgeons in attendance, who, in consequence of the inflamed state of the leg, dreaded gangrene of the stump coming on. The operation was however performed early next morning. The edges of the wound were simply approximated, and kept so by means of lint smeared with a layer of styrax ointment: this position was facilitated by my having made, during the operation, two perpendicular incisions, one over the crest of the tibia and the other on the opposite side. *The first dressing was not removed for nine days*, and then the wound was found to be suppurating freely: it was entirely healed by the 31st day after the operation.

General *Dulong* received a gun-shot wound in the right axilla in the Polish campaign of 1807. The ball had passed through the tendon of the pectoral muscle, and the plexus of nerves, grazing the shoulder-joint. Although there was scarcely any hæmorrhage, there was good reason to believe that the axillary artery had been divided. Some years subsequently this officer called upon me for my advice. I then learned that, immediately upon the receipt of the wound, the extremity had been stricken with a paralytic torpor and a sense of most distressing coldness, and that no pulse could be felt at the wrist. It had been unwisely determined to try to save the limb. The consequence was that the hand and arm remained quite paralysed, and became so atrophied as to resemble a part of a mummy rather than of a living man. I recommended him to submit to amputation; but he would not. His infirmities increasing, he committed suicide.

Marshal *Duroc*, Duke of Friuli, was wounded in the right groin, by a piece of a shell at St. Jean d'Acre. At the close of the battle of Wurtschen, 1813, a cannon-ball, which had cut the body of General *Kirschner* (who was riding at his side) in twain, grazed him in the belly. A large portion of the abdominal parietes was carried away, and the bowels wounded in several places. He survived about thirty hours in dreadful agony.

General *Foy* was under my care in his last illness, the disease being cancer of the stomach. " Had his medical attendants made use of the moxa as I recommended, his life might probably have been saved, as I have succeeded in doing so in not a few cases in private practice."

General Baron *Ganan* was wounded at the battle of Dresden in the occiput. It was necessary to apply the trephine to remove some portions of the depressed bone: ultimately he recovered. " A phenomenon, little known to medical men, was often noticed by us in this case. When the ear was applied to the cicatrix over the perforation, the *bruissement* of the cerebral arteries was distinctly perceptible; and the General himself could hear the sounds of the voice when directed to the seat of the wound—a circumstance that still continues, and therefore clearly shews that the opening in the cranium is not yet entirely closed." (Is there anything wonderful in this?)

Marshal *Grouchy* received several severe contused wounds in the thighs and legs at the battles of Moskowa and Craoune. In the case of his wife, *Madame la Marechale*, I excised the left mamma for a cancerous tumor. This lady quite recovered; she subsequently died very suddenly from a sort of spasmodic cholera, or acute neurosis.

General *Kleber* received, at the capture of Alexandria, a ball that struck him in the right temple, divided the integuments, and grazed the parietal bone. I dressed the wound at the base of Pompey's Pillar, and he was sent on to Alexandria. He was subsequently killed at the battle of *Heliopolis*, where I was exposed to the greatest danger in the discharge of my professional duties.

Marshal *Lannes*, Duke of Montebello, was struck by a musket-ball in the right leg, at the battle of Aboukir. Symptoms of tetanus threatened to come on, but were fortunately subdued. He returned with his colleague *Murat* to France, a few weeks after General *Buonaparte*. He had been wounded also in the temple at the thirteenth assault of St. Jean d'Acre. Some years afterwards he received several wounds in various engagements in Spain. At the famous battle of Essling (1809) his right leg was shattered by a cannon-ball, which passed through the knee-joint and wounded the flesh of the left thigh. Immediate amputation was performed by me; and he was sent on to Ebersdorf, where he caught the typhus fever, which prevailed at that time in the army: he died on the 13th day after the battle.

General *Lawless*—once a Professor of Physiology in Dublin!—who commanded the third foreign regiment, consisting almost entirely of Irishmen, had his left leg shot away by a cannon-ball at Lovemberg, on the frontiers of Bohemia. I amputated the limb immediately below the head of the tibia; and, as the army were retreating back upon Dresden at this time, I advised my patient to ride direct to his own home in France, without doing anything to the dressings of his wound, except sponging their surface daily, and keeping the stump enveloped in a piece of linen-cloth, or a sheep's-skin. By following my directions, he rode on horseback the entire distance from the scene of action to his residence in Tours, having his stump all the while suspended by a belt that passed over his shoulders, and without having it dressed once. On removing the apparatus, when he reached his journey's end, the wound was nearly healed. As a mark of his gratitude, the General made me a present of a magnificent English engraving of *Hunter*.

Baron *Menou*, the third in command of the Egyptian army, was seized with sporadic Plague, just before our debarkation to France: already there were three carbuncles on the right leg, and an incipient one had appeared in the right groin. Unwilling to make the Captain of the English frigate (Dido)—which was to convey us home—acquainted with the real facts, I had the General strictly confined to his own cabin, and I then excised the carbuncles,* administering internally camphor, nitre, bark, and laudanum. The wounds suppurated freely, before they healed. He was completely cured, when we reached Toulon.

General Viscount *Mermet*, in consequence of a fall upon his loins in one of the last actions in Italy under Prince *Eugene*, became entirely paraplegic; the palsy being accompanied with incontinence of the urine. Cupping in the first instance, and subsequently the repeated (20 times) application of moxas, restored him to perfect health, so that he was able to resume active service.

General *Mireur*, after having been wounded in the right shoulder at the glorious battle of the Pyramids, was murdered a few days afterwards by the Arabs, while conveying the orders from *Buonaparte* to Admiral *Brueys*, to weigh anchor and proceed direct to Corfu, for the purpose of taking in fresh troops and bringing them to Egypt. The death of this young officer was the cause of the destruction of our fleet at Aboukir, and of our subsequent loss not only of Malta, but also of Egypt, which would otherwise have become one of the richest and most beautiful of French colonies.

Marshal *Moncey*, Duke of Cornegliano, became affected, in 1830, with an enormous hydrocele. I performed an operation and effected a radical cure.

General *Netherwoot*, in one of the combats in Syria, received, from the hands of one of the Mamelukes, a sabre-cut in the right thigh: the entire thickness of the extensor muscles was divided down to the bone, and even this was deeply notched. The wound was very large as well as deep. While one of my assistants kept the limb completely extended, I passed ten stitches from the bottom of the wound through the flesh and integuments, so that the edges might be retained in exact apposition throughout the entire depth. After being bandaged, the limb was placed in a fracture-box, and maintained in a state of extension. This excellent officer was subsequently killed in the expedition to St. Domingo.

General Count *Pajol* was wounded in the left fore-arm at the battle of Moskowa. As both bones were broken, and the soft parts much injured, many surgeons would unquestionably have recommended immediate amputation; but I determined to try to save the limb. After having freely *debridé* the wound, and removed all the fragments of bones, I brought the edges together, and applied my apparatus, which I rendered immovable, the fore-arm being kept bent and suspended in a sling. This distinguished warrior accompanied the march of the troops during the dreadful retreat from Russia; and, although the wound was not dressed above five or six times, it eventually cicatrised completely, without the movements of the limb being at all impeded.

General *Silly* was wounded by a cannon-ball in the right knee, at the second battle of Aboukir. I had scarcely completed the amputation of the limb, when a charge of the English cavalry came down upon us. I took the wounded man upon my shoulders, and carried him along a very uneven

---

* This appears to us very extraordinary practice; and yet the Baron continued in after-life to be so convinced of its propriety that—as we have seen in a preceding page—he actually recommended its adoption to the physicians in Dublin.

road, to the rear-guard of our army. I arrived at Alexandria with my honorable burden, and had the satisfaction there of completing the cure.

Marshal *Soult* was never dressed by me on the field of battle; but I attended him in Paris for a severe contusion of the leg, which had been fractured. A traumatic sanguineous tumor supervened: this required a delicate operation should be performed. The success was complete; and my illustrious patient speedily recovered.

Marshal *Suchet*, Duke of Albufera, on returning from his most arduous campaigns in Spain, consulted me for a cancerous affection of the stomach, the precursory symptoms of which had already appeared. The disease, although it had been very sensibly arrested, finally proved fatal.

Marshal *Victor*, Duke of Belluno, was wounded in the right thigh, at the battle of Craoune (1814). The ball had passed between the femur and the femoral artery, which I found to be partly denuded: the sciatic nerve was injured. The two wounds were immediately 'debridées,' and subsequently dressed after my usual plan. The Marshal continued to suffer from a traumatic neuralgia to the end of his life, in spite of every remedy that was tried.

General *Zayonchek*, 75 years old, was wounded in the knee at the passage of the Beresina. The wound required the amputation of the limb. I performed the operation under the cannon of the enemy, and while there was a very heavy fall of snow. To prevent my being incommoded by 'ce meteore,' two general officers held the patient's cloak over our heads. He returned to Warsaw, his native place, and finally recovered. He was subsequently made a prince, and the Viceroy of Poland by Emperor Alexander: he died at the advanced age of 86 years.

As frequent allusion has been made, in the preceding pages, to the 'appareil inamovible' used by our author in his military service, it may not be unprofitable briefly to describe it here, although we have given an account of it some years ago in this Journal. One or two prefatory remarks may be made with advantage.

*Larrey* is a great enemy to the frequent dressing of all recent wounds, whether these be simple or complicated with fracture of bones. In the case of gun-shot wounds, the frequent renewal of the dressings is, we need scarcely say, often quite impracticable; it is therefore a matter of serious importance to know the best mode of proceeding under such circumstances. The authority of so experienced a writer as our author will very properly command no ordinary attention. The main object of his instructions seems to be, to place the wound in such a position that Nature is interfered with as little as possible in the reparation of the injury, that has been inflicted. With this view he seeks to maintain the part in perfect quietude, guarding it from exposure to the outward air, and maintaining a proper temperature around it; and effecting these ends, without confining the patient to bed, or even to the house. He found by experience that in cold seasons wounds were, on the whole, more tardy of healing—especially if they were frequently exposed to the air—than in warm ones. He had too often occasion to verify the truth of this remark in the dreadful campaigns of Russia, Saxony and France; especially when contrasted with the memorable one of Egypt. It was during these wars that the admirable effects of his 'appareil inamovible' were most conspicuous. We were retreating, he says, all the while, and yet unwilling to leave our wounded behind. Accordingly, most of our patients, after they had their wounds dressed according to my plan, were immediately dispatched forwards, and some of them even reached their homes in France before the original dressings of their wounds were removed. We give but one instance. A soldier, after having his arm amputated at the shoulder-joint after the terrible battle of Moskowa, was advised to proceed direct on to his native place in Provence, and do nothing to the wound except keep it clean by sponging away any discharge from the outward dressings, and cover the limb with a good sheep's-skin, to guard it from the cold and damp air. When he reached home, and the dressings were removed, the wound was found to be nearly cicatrized. This is one of many scores of similar cases, where the primary dressings were not removed for two, three, or even four weeks after they had been put on.

The following are the instructions given by *Larrey* how to arrange and apply his " appareil." If the fracture of a limb be complicated with a wound, we should first simplify it, as much as possible, by freely incising (*debrider*) its edges, extracting all foreign substances or pieces of broken bone, and arresting the hæmorrhage. When all has been done, we should approximate the edges and keep them in apposition by means of a piece of linen (linge fenetré) on which some balsamic substance, such as the Styrax ointment, balsam of Mecca, &c., has been spread. Pledgets of lint, carded cotton or tow, are then to be placed over the dressing and the anfractuosities that correspond to the wound; also, some square compresses dipped in a glutinous strengthening fluid, that is prepared by beating up the white of two or three eggs with camphorated wine or vinegar, or, in lieu of these, with salt water. These compresses should be carefully applied over the whole of the injured part, while an assistant is engaged in maintaining the fractured bone or bones in exact apposition by appropriate extension and counter-extension. These compresses perform the service of immediate splints, without their inconvenience. They are kept in their place and are moreover strengthened by an eighteen-tailed bandage, which, when properly applied, maintains every thing firmly together. The foot and ankle-joint should previously be enveloped in long compresses, that have been wetted in the same fluid. A pad of tow, of a pyramidal shape, is to be placed under the *tendo Achillis*, to make all level and even, so that the pressure of the apparatus my be uniform throughout. The surgeon is then to take two cylindrical rolls of new rye straw, and, wrapping them up in the opposite edges of a towel or small sheet, which is to be stretched out under the leg, he applies them on each side, having previously interposed two or three flat cushions filled with oat-chaff, to prevent the pressure of the straw rolls on the skin. These are secured in their place by a good many broad tapes or ribbons, which should be tied over the outer roll of straw, and in such a manner that they do not press directly upon the crest of the tibia. The advantage of the straw rolls is that they are so elastic, that, although they give way at first to the swelling of the limb, they continue to remain in close contact with it as the swelling subsides, and no void ever exists between it and the apparatus—an objection to the paste-board splints, and other substitutes which have been suggested of late years. As a substitute for the foot-board, I use a piece of a folded sheet placed like a stirrup under the sole, some tow being previously interposed to fill up the concavity; the two ends are brought up, crossed over the instep, and then secured to the straw rolls with strong pins.

The loose end of the sheet, stretched under the leg, which extends below the inferior ends of the straw-rolls, is to be folded under the foot and over the ends of the rolls, and then firmly secured by a few stitches or pins.

With such a simple, and yet so efficient, an apparatus, the reader will readily understand how a patient is able to walk about with the aid of crutches, the foot being merely suspended by a ribbon passed over the neck. The same apparatus is equally well suited for fractures of the body, or of the neck, of the femur. We have only to lengthen the external straw-roll as far up as the pelvis, and secure it firmly by a belt passed round the body. The limb, in such cases, should be kept in the horizontal position, until the callus has become consolidated.

The camphorated albumino-vinous wash, which we have recommended, has the great advantage of increasing the tonic action of the injured parts, and of preserving their natural heat, without obstructing the meshes of the linen or cotton cloth that is used in making the apparatus. The thinner portion of the discharge oozes out, and may be removed with a sponge, whenever it is necessary.

